# Abdominal multi-organ iron content and the risk of Parkinson’s disease: a Mendelian randomization study

**DOI:** 10.3389/fnagi.2024.1416014

**Published:** 2024-08-14

**Authors:** Mingrui Yang, Cheng Tang, Fei Peng, Chaotian Luo, Guowei Chen, Rong Kong, Peng Peng

**Affiliations:** ^1^Department of Radiology, The First Affiliated Hospital of Guangxi Medical University, Nanning, Guangxi, China; ^2^NHC Key Laboratory of Thalassemia Medicine, Guangxi Medical University, Nanning, Guangxi, China

**Keywords:** abdominal multi-organ, iron content, Parkinson’s disease, iron metabolism, Mendelian randomization

## Abstract

**Background:**

To evaluate the causal relationship between abdominal multi-organ iron content and PD risk using publicly available genome-wide association study (GWAS) data.

**Methods:**

We conducted MR analysis to assess the effects of iron content in various abdominal organs on PD risk, followed by reverse analysis. Additionally, MVMR analysis evaluated the independent effects of organ-specific iron content on PD. We utilized genetic variation data from the UK Biobank, including liver iron content (*n* = 32,858), spleen iron content (*n* = 35,324), and pancreas iron content (*n* = 25,617), as well as summary-level data for Parkinson’s disease from the FinnGen (*n* = 218,473) and two other large GWAS datasets of European populations (First dataset *n* = 480,018; Second dataset *n* = 2,829). The primary MR analysis used the inverse variance-weighted (IVW) method, confirmed by MR-Egger and weighted median methods. Sensitivity analysis was performed to address potential pleiotropy and heterogeneity. Observational cohort results were validated through replication cohort analysis, followed by meta-analysis.

**Results:**

IVW analysis revealed a causal relationship between increased liver iron content and elevated risk of PD (OR = 1.27; 95% CI: 1.05–1.53; *p* = 0.015). No significant causal relationship was observed between spleen (OR = 1.00; 95% CI: 0.76–1.32; *p* = 0.983) and pancreatic (OR = 0.93; 95% CI: 0.72–1.20; *p* = 0.573) iron content and increased risk of PD. Meta-analysis of GWAS data for PD from three different sources using the random-effects IVW method showed a statistically significant causal relationship between liver iron content and the occurrence of PD (OR = 1.17, 95% CI: 1.01–1.35; *p* = 0.012).

**Conclusion:**

This study presents evidence from Mendelian randomization (MR) analysis indicating a significant causal link between increased liver iron content and a higher risk of Parkinson’s disease (PD). These findings suggest that interventions targeting body iron metabolism, particularly liver iron levels, may be effective in preventing PD.

## 1 Introduction

Parkinson’s disease (PD) is the second most common neurodegenerative disease globally (Collaborators GBDP, 2018), severely impacting patients’ quality of life. The pathogenesis of PD is multifactorial, potentially involving genetic factors, oxidative stress, and immune dysregulation (Mollenhauer and von Arnim, 2022). Research has shown a notable increase in iron content in the basal ganglia of PD patients, which correlates with symptom severity (Thomas et al., 2020). Conversely, decreased iron levels in the temporal cortex and increased iron deposition in the substantia nigra have also been reported (Mochizuki et al., 2020). Iron is crucial for brain function (Wang et al., 2016), yet its excessive accumulation can lead to oxidative stress and neuronal damage, contributing to neurodegenerative processes (Guan et al., 2017). Therefore, abnormal brain iron deposition is implicated in PD development and progression.

The liver, spleen, and pancreas play crucial roles in iron metabolism in the body. These three organs work closely together to maintain the body’s iron balance and ensure normal physiological function. The liver serves as the primary iron storage and regulator. Hepatocytes store and regulate iron in the body through ferritin and other proteins (Nemeth and Ganz, 2021). Hepatocytes in the liver can release and store iron, dynamically balancing it according to the body’s needs to maintain normal iron levels during metabolism and synthesis processes. The spleen also plays a critical role in iron metabolism. It is responsible for clearing aged red blood cells and recycling iron elements from them (Wang and Babitt, 2016). After the red blood cells in the spleen rupture, iron is released and stored in the spleen, while also undergoing some regulatory iron metabolism processes. The pancreas has multiple functions in body iron metabolism. It not only participates in iron storage and release but also regulates insulin synthesis and secretion. Iron is a necessary component of insulin synthesis, making the pancreas essential for maintaining normal blood sugar levels and energy metabolism (De Domenico et al., 2006). Recent research progress highlights the complex interactions of iron metabolism in the liver, spleen, and pancreas, and the imbalance of iron in these organs may be associated with various diseases such as liver disease, anemia, and metabolic disorders (Soares and Weiss, 2015).

Despite increasing evidence suggesting that abnormal iron metabolism can lead to pathological changes in the nervous system (David et al., 2022), some studies have found a lack of increased iron concentration in patients with early-stage Parkinson’s disease (Bergsland et al., 2019), suggesting that iron deposition is not the initial event of PD. Additionally, previous observational studies cannot explain the role of confounding factors in the disease process, and whether changes in iron content in multiple abdominal organs are directly related to the occurrence of Parkinson’s disease remains unclear. Therefore, it is crucial to directly explore the assumed causal effect of iron content in multiple abdominal organs on Parkinson’s disease, and vice versa. Here, we conducted a Mendelian randomization (MR) study to further evaluate whether liver iron content, spleen iron content, and pancreatic iron content affect the risk of Parkinson’s disease.MR uses single nucleotide polymorphisms (SNPs) as genetic variants, making MR designs highly reliable, avoiding the influence of reverse causality and potential confounding factors in observational studies (Sekula et al., 2016). Therefore, MR is widely used to examine causal relationships between exposures and clinical outcomes (Richmond and Davey Smith, 2022). This study not only contributes to understanding the pathogenesis of Parkinson’s disease related to iron deposition in the body but also provides new important clues for early prevention of Parkinson’s disease, optimizing clinical management strategies for Parkinson’s disease patients.

## 2 Materials and methods

### 2.1 Study design

**Figure 1:**
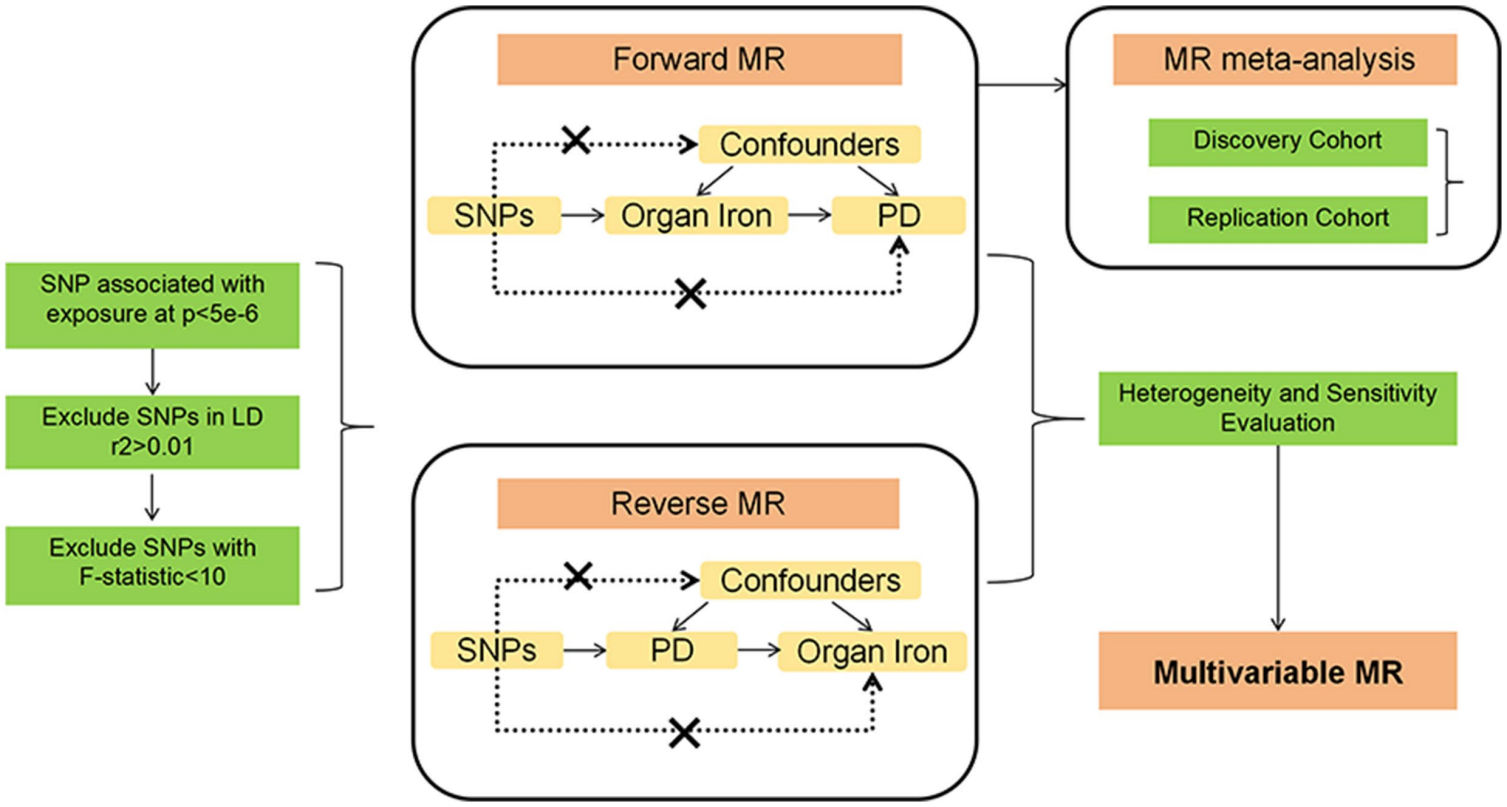
The MR framework of ideation for our study.

This study employed two-sample Mendelian randomization (MR) analyses to investigate the causal relationship between abdominal multi-organ iron content and Parkinson’s disease (PD). We used genetic variants as instrumental variables (IVs) for exposures (iron content in liver, spleen, and pancreas) and outcomes (PD risk). To ensure the reliability of MR results, MR analysis needs to meet three main assumptions (Boef et al., 2015): (1) IVs are strongly associated with exposure; (2) IVs are not linked to confounders; (3) IVs influence the outcome only through the exposure. We utilized datasets entirely based on European populations to reduce selection bias due to different ethnicities and thereby enhance the robustness of the analysis. This study fully utilized publicly available GWAS data, thus no specific ethical approval was required for this study. All details regarding ethical approvals for each study and participant informed consent in the GWAS datasets can be found in the corresponding records in the original publications. A conceptual MR framework is illustrated in Figure 1.

### 2.2 Data source

In this study, we obtained summary data from genome-wide association studies (GWAS) related to iron content in multiple organs from the UK Biobank. This dataset includes 32,858 participants with liver iron content, 25,617 participants with spleen iron content, and 35,324 participants with pancreatic iron content, all of British ancestry. Each of the three exposure datasets contains approximately 9.27 million SNPs.

As for the discovery analysis cohort, Parkinson’s disease GWAS data were obtained from the FinnGen database, utilizing data annotated with strictly defined Parkinson’s disease phenotypes, comprising 1,843 cases and 216,630 controls. The Parkinson’s disease dataset for the analysis cohort was released in 2021. The primary reason for using this version was the lack of updates in GWAS data with phenotypes annotated as strictly defined Parkinson’s disease in the latest version available to us. As for the replication analysis cohort, Parkinson’s disease GWAS data were obtained from two previously published related genome-wide association studies, both conducted on European populations: the first replication sample’s Parkinson’s disease dataset was derived from a GWAS study conducted by Sakaue et al., 2021 including 2,638 cases and 477,380 controls, with approximately 24.91 million SNPs (Sakaue et al., 2021); the second replication sample’s Parkinson’s disease dataset was derived from a GWAS study conducted by Alfradique-Dunham et al., 2021 including 1,570 cases and 1,259 controls, with approximately 12.86 million SNPs (Alfradique-Dunham et al., 2021). The corresponding data compositions are displayed in Table 1.

**Table 1:** Details of the GWASs included in the Mendelian randomization.

Consortium/Dataset	Phenotype	Sample size	SNP(n)	Population
UK Biobank	Liver iron	32,858	9,275,407	European
UK Biobank	Spleen iron	35,324	9,275,407	European
UK Biobank	Pancreas iron	25,617	9,275,407	European
FinnGen	PD	218,473	16,380,461	European
GWAS by Sakaue et al. (2021)	PD	480,018	24,194,622	European
GWAS by Alfradique-Dunham et al. (2021)	PD	2,829	12,858,066	European

SNP, single nucleotide polymorphism.

### 2.3 Single nucleotide polymorphism selection

Firstly, liver iron content, spleen iron content, and pancreatic iron content were each considered as exposures, and SNPs with genome-wide significance threshold (*p* < 5 × 10^−6^) were selected as instrumental variables to investigate their impact on prognosis. To ensure that the selected instrumental variables were independent, SNPs with linkage disequilibrium (*r*^2^ > 0.01, kb < 10,000) were removed. Additionally, SNPs with inconsistent alleles and those with ambiguous strand palindromes were excluded. To prevent bias from weak instrumental variables, the strength of the selected SNPs was measured using the *F*-statistic, with an *F*-statistic greater than 10 indicating a strong instrument (Georgakis et al., 2019). In this study, the calculation formula for the *F*-statistic is: *F*-statistic = *R*^2^ × (N – 2)/(1 – *R*^2^), where *R*^2^ = 2 × EAF × (1 – EAF) × *β*^2^. We found that the statistical range of all SNPs exceeded 30, indicating that they were strong instruments for MR analysis. SNPs achieving genome-wide significance (*p* < 5 × 10^−6^) in the genome-wide association study (GWAS) were selected to ensure a significant association with the exposures. SNPs with *r*^2^ > 0.01 and within 10,000 kb of each other were excluded to ensure the independence of the selected SNPs and to reduce potential collinearity. Additionally, SNPs with inconsistent alleles and ambiguous strand palindromes were excluded to avoid errors or inconsistencies in genotype data. The F-statistic was calculated for each SNP to assess its strength as an instrumental variable, with a threshold of *F* > 10 indicating strong instruments. These detailed selection and validation procedures ensure the reliability and validity of the instrumental variables used in the study, thereby enhancing the robustness and scientific value of the findings.

### 2.4 Data analysis

Initially, the two-sample MR package was employed to estimate the causal relationship between exposure and outcome. Bidirectional MR analysis was performed on the discovery sample, followed by assessing heterogeneity and pleiotropy in the analysis results. Considering the clinical correlations among iron content in multiple abdominal organs, Multivariable MR (MVMR) analysis was conducted to evaluate their independent effects on Parkinson’s disease (Sanderson et al., 2019). For the discovery cohort with statistically significant differences in results, further validation was conducted using the replication cohort. Finally, the analysis results of the discovery cohort and the replication cohort were combined for meta-analysis.

In the bidirectional MR analysis, the inverse variance weighted (IVW) method was employed as the primary statistical model (Burgess et al., 2015). A *p*-value <0.05 was considered statistically significant, indicating a significant causal relationship, and odds ratios (OR) with 95% confidence intervals (CI) were reported for the MR estimates. Additionally, as a supplement, sensitivity analyses based on the weighted median (WM) method (Hartwig et al., 2017) and MR-Egger method (Bowden et al., 2015) were conducted to ensure the robustness of instrumental variable pleiotropy. A *p*-value <0.05 was considered statistically significant in these sensitivity analyses. Sensitivity analyses were conducted to evaluate heterogeneity and pleiotropy using Cochran’s Q heterogeneity test, Egger intercept test, MR pleiotropy residual sum and outlier (MR-PRESSO) global test, and leave-one-out test to ensure the stability of the results. A *p*-value less than 0.05 for Cochran’s Q indicated significant heterogeneity (Greco et al., 2015). In the estimation of causal effects, directional horizontal pleiotropy was indicated by the intercept in MR-Egger regression (Bowden et al., 2015). MR-PRESSO was also used to identify and correct for outliers within the association (Verbanck et al., 2018). The leave-one-out test sequentially excluded each SNP and conducted MR analysis on the remaining SNPs to detect potential outlier instrumental variables (Hemani et al., 2018). Results for each SNP and the final MR results can be illustrated using forest plots.

MVMR analysis utilized the IVW method, with a *p*-value <0.05 considered statistically significant, indicating a significant causal relationship, and OR with 95% CI reported for MVMR estimates.

All steps for instrumental variable selection and quality control were conducted using the software packages TwoSampleMR (v 0.5.8), Mendelian Randomization (0.9.0), TwoSampleMR (0.5.8), MR-PRESSO (1.0), MVMR (0.4), and meta (6.5–0) in R language version 4.3.2.

## 3 Results

### 3.1 Initial MR analysis

We conducted a two-sample Mendelian Randomization (MR) analysis using summary statistics from the FinnGen consortium to evaluate the causal effects of iron content in the liver, spleen, and pancreas on the risk of Parkinson’s Disease (PD). The primary MR analysis utilized the inverse variance weighted (IVW) method. Our findings indicated a significant causal relationship between liver iron content and an increased risk of PD (IVW: OR = 1.27; 95% CI: 1.05–1.53; *p* = 0.015). This suggests that higher iron levels in the liver may contribute to the development of PD, which aligns with previous research indicating the role of iron in neurodegenerative processes.

In contrast, no significant causal relationship was observed between spleen iron content (IVW: OR = 1.00; 95% CI: 0.76–1.32; *p* = 0.983) or pancreatic iron content (IVW: OR = 0.93; 95% CI: 0.72–1.20; *p* = 0.573) and the risk of PD. These results imply that the iron content in these organs does not significantly influence PD risk, highlighting the specificity of liver iron in this context.

To further validate our findings, we conducted a reverse MR analysis. This analysis confirmed that there was no significant causal relationship between PD and liver iron content (IVW: OR = 1.00; 95% CI: 0.97–1.02; *p* = 0.861), spleen iron content (IVW: OR = 0.98; 95% CI: 0.84–1.00; *p* = 0.111), or pancreatic iron content (IVW: OR = 1.00; 95% CI: 0.97–1.03; *p* = 0.828). This suggests that while liver iron content may influence PD risk, the reverse is not true, thus reinforcing the directionality and potential causality of our initial findings.

**Figure 2:**
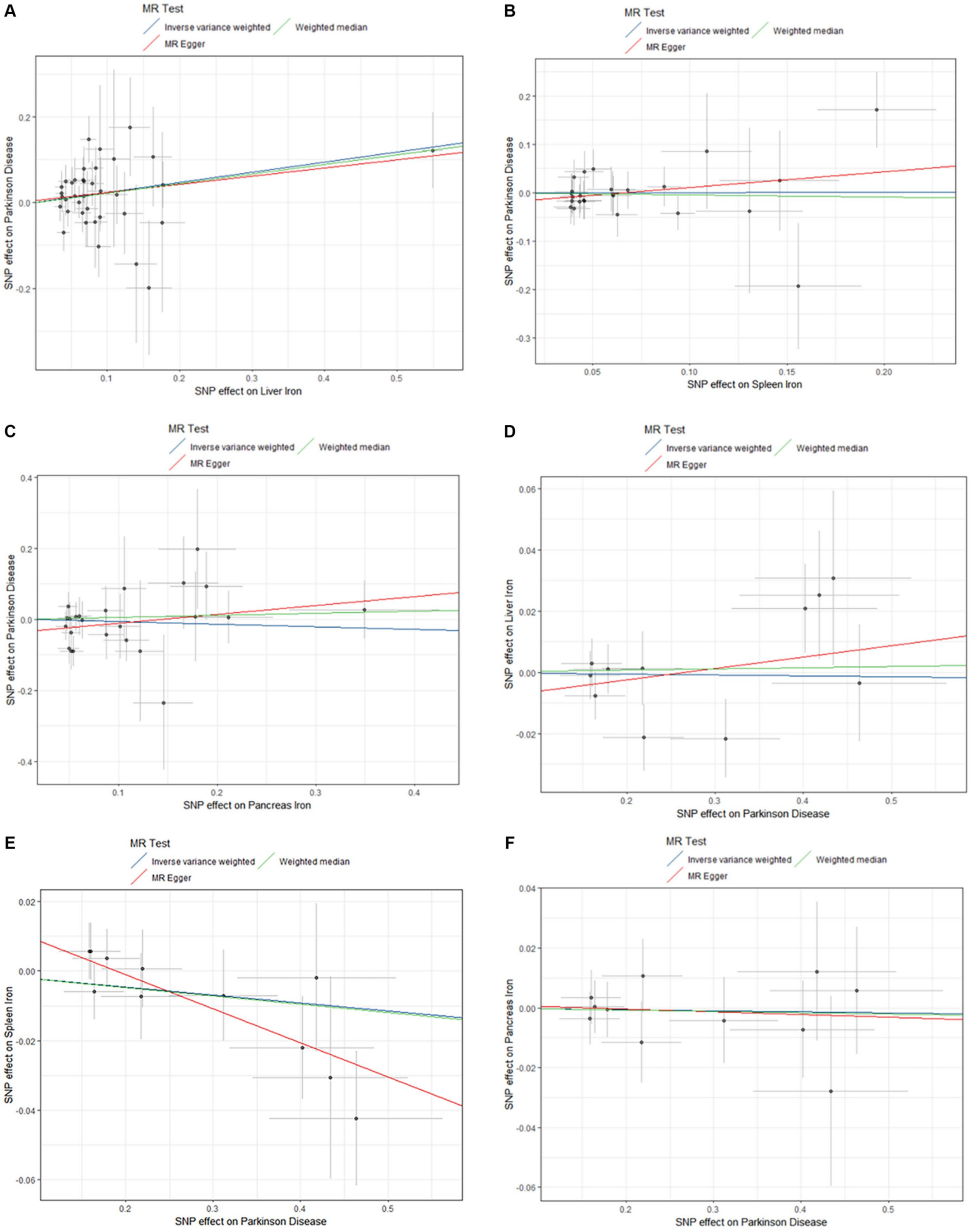
Scatter plots of primary MR analysis. The slope of each line represents the expected MR impact in various models. **(A)** Liver iron on PD; **(B)** Spleen iron on PD; **(C)** Pancreas iron on PD; **(D)** PD on Liver iron; **(E)** PD on Spleen iron; **(F)** PD on Pancreas iron. MR, Mendelian randomization; PD, Parkinson’s disease.

A comprehensive overview of these results is presented in Table 2 and Figure 2.

**Table 2:** The result of MR study and reverse MR study.

Exposure	Outcome	Method	SNP (*n*)	*β*	se	*p*-value	OR (95CI%)
Liver iron	PD	Inverse variance weighted	37	0.236	0.097	0.015	1.27 (1.05–1.53)
		MR Egger	37	0.190	0.154	0.224	1.21 (0.89–1.64)
		Weighted median	37	0.223	0.144	0.121	1.25 (0.94–1.66)
Spleen iron	PD	Inverse variance weighted	25	0.003	0.141	0.983	1.00 (0.76–1.32)
		MR Egger	25	0.322	0.337	0.349	1.38 (0.71–2.67)
		Weighted median	25	−0.045	0.204	0.826	0.96 (0.64–1.43)
Pancreas iron	PD	Inverse variance weighted	24	−0.072	0.128	0.573	0.93 (0.72–1.20)
		MR Egger	24	0.250	0.216	0.258	1.28 (0.84–1.96)
		Weighted median	24	0.057	0.200	0.776	1.06 (0.71–1.57)
PD	Liver iron	Inverse variance weighted	11	−0.003	0.016	0.861	1.00 (0.97–1.03)
		Inverse variance weighted	11	0.037	0.042	0.397	1.04 (0.96–1.13)
		Inverse variance weighted	11	0.004	0.021	0.849	1.00 (0.96–1.05)
PD	Spleen iron	Inverse variance weighted	11	−0.023	0.014	0.111	0.98 (0.95–1.01)
		Inverse variance weighted	11	−0.098	0.03	0.03	0.91 (0.84–0.98)
		Inverse variance weighted	11	−0.024	0.02	0.233	0.98 (0.94–1.02)
PD	Pancreas iron	Inverse variance weighted	11	−0.003	0.016	0.828	1.00 (0.97–1.03)
		Inverse variance weighted	11	−0.009	0.042	0.832	0.99 (0.91–1.08)
		Inverse variance weighted	11	−0.004	0.020	0.827	1.00 (0.96–1.04)

OR, odds ratio; CI, confidence interval; MR, Mendelian randomization; NA, not available. The statistically significant difference with a *p*-value less than 0.05 (*p* < 0.05).

### 3.2 Multivariable MR (MVMR) analysis

To explore the independent effects of iron content in different abdominal organs on PD risk, we performed a Multivariable MR (MVMR) analysis. The MVMR analysis indicated that liver iron content remained significantly associated with an increased risk of PD (IVW: OR = 1.23; 95% CI: 1.03–1.47; *p* = 0.025). This reinforces the importance of liver iron in the pathogenesis of PD.

However, the analysis revealed no significant causal relationships for spleen iron content (IVW: OR = 0.90; 95% CI: 0.72–1.12; *p* = 0.656) or pancreatic iron content (IVW: OR = 0.95; 95% CI: 0.74–1.21; *p* = 0.337) with PD risk. These findings suggest that, independently, neither spleen nor pancreatic iron levels significantly impact PD risk, further emphasizing the unique role of liver iron content.

The results of MVMR analysis are presented in Table 3.

**Table 3:** The results of MVMR study.

Exposure	Outcome	Method	SNP (*n*)	β	se	*p*-value	OR (95CI%)
Liver iron	PD	Inverse variance weighted	85	0.205	0.091	0.025	1.23 (1.03–1.47)
Spleen iron	PD	Inverse variance weighted	85	−0.056	0.126	0.656	0.95 (0.74–1.21)
Pancreas iron	PD	Inverse variance weighted	85	−0.110	0.115	0.337	0.90 (0.72–1.21)

MVMR, multivariable Mendelian randomization; OR, odds ratio; CI, confidence interval; SNPs, single nucleotide polymorphisms; NA, not available. The statistically significant difference with a *P*-value less than 0.05 (*P* < 0.05).

### 3.3 Replication analysis

For replication, we utilized summary statistics from two other large GWAS datasets. The replication analysis results from the GWAS conducted by Sakaue et al. (2021) (IVW: OR = 1.06; 95% CI: 0.93–1.22; *p* = 0.387) and the GWAS by Alfradique-Dunham et al. (2021) (IVW: OR = 1.30; 95% CI: 0.93–1.82; *p* = 0.121) did not show significant causal relationships between liver iron content and PD. However, a meta-analysis combining these datasets with the FinnGen data suggested a significant association between increased liver iron content and a higher risk of PD (OR = 1.17; 95% CI: 1.01–1.35; *p* = 0.012). This meta-analysis reinforces our initial findings and highlights the robustness of the association between liver iron and PD risk across different populations.

**Figure 3:**
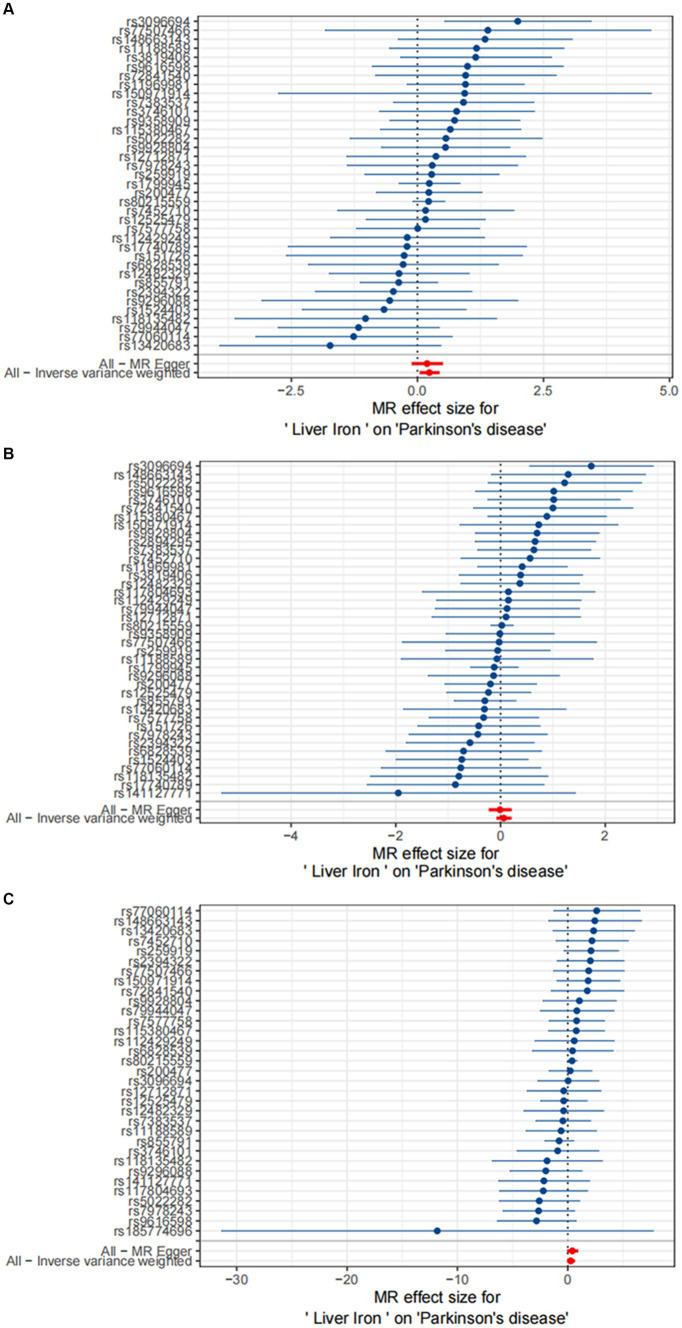
Causal effects of Liver iron on PD. **(A)** The GWAS data for Parkinson’s disease is derived from FinnGen; **(B)** The GWAS data for Parkinson’s disease is derived from the summary statistics published by Sakaue et al. (2021); **(C)** The GWAS data for Parkinson’s disease is derived from the summary statistics published by Alfradique-Dunham et al. (2021).

**Figure 4:**
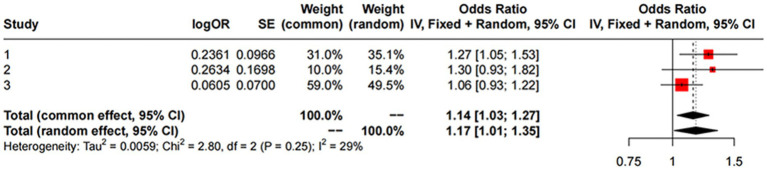
MR meta-analysis results between Liver Iron on PD. Study 1:Date from FinnGen; Study 2:Date from GWAS by Sakaue et al. (2021); Study 3:Date from GWAS by Alfradique-Dunham et al. (2021).

The results are shown in Figures 3, 4.

### 3.4 Sensitivity analyses

To ensure the robustness of our findings, we conducted several sensitivity analyses. These analyses included heterogeneity tests using Cochran’s *Q* and assessments of horizontal pleiotropy using the MR-Egger intercept test. The Cochran’s *Q* test indicated no significant heterogeneity across all analyses, suggesting consistent results. Additionally, the MR-Egger intercept test did not reveal any evidence of horizontal pleiotropy, further supporting the validity of our causal estimates. These comprehensive sensitivity analyses confirm the reliability and robustness of our findings, particularly the significant association between liver iron content and PD risk.

The results of sensitivity analysis are presented in Table 4.

**Table 4:** The results of sensitivity analyze.

Exposure	Outcome	SNP (*n*)	Heterogeneity test		MR-Egger pleiotropy test		MR-PRESSO results		
			*Q*	*p*-value	Intercept	*p*-value	RSSobs	*p*-value	Outlier
Liver iron	PD (FinnGen)	37	31.553	0.680	0.005	0.705	32.702	0.708	NA
Liver iron	PD (GWAS by Sakaue et al. (2021))	40	39.257	0.458	0.009	0.383	40.648	0.495	NA
Liver iron	PD (GWAS by Alfradique-Dunham et al. (2021))	33	31.601	0.487	−0.020	0.373	33.908	0.509	NA
Spleen iron	PD (FinnGen)	25	16.057	0.886	−0.021	0.308	18.646	0.846	NA
Pancreas iron	PD (FinnGen)	24	20.130	0.634	−0.036	0.076	21.648	0.675	NA
PD (FinnGen)	Liver iron	11	12.524	0.252	−0.010	0.330	15.463	0.233	NA
PD (FinnGen)	Spleen iron	11	7.929	0.636	0.019	0.063	9.797	0.607	NA
PD (FinnGen)	Pancreas iron	11	3.158	0.977	0.001	0.886	3.692	0.982	NA

The statistically significant difference with a *P*-value less than 0.05 (*P* < 0.05).

## 4 Discussion

In this MR study utilizing summary statistics data from FinnGen and two other large European ancestry GWAS datasets, we investigated the causal relationship between iron content in multiple abdominal organs and Parkinson’s disease. Our study findings revealed a positive correlation between genetically predicted liver iron content and the risk of Parkinson’s disease. These findings provide novel insights into the potential role of monitoring iron levels in specific organs for preventing and treating Parkinson’s disease and mitigating its complications.

Iron is an extremely important trace element in the human body, playing a crucial role as a cofactor in various biological processes. In the brain, iron is involved in multiple processes such as neurotransmitter and myelin synthesis, mitochondrial respiration, and neurotransmitter transmission. The brain, being the organ with the highest oxygen consumption in the body, relies on iron ions for electron transport in mitochondria to generate adenosine triphosphate (ATP) (Ferreira et al., 2019), which serves as energy for the brain. Additionally, iron ions participate in the generation of Fe–S clusters, which are involved in protein repair within the mitochondrial respiratory chain (Yang et al., 2023). Prolonged elevation of iron ion levels beyond the maximum binding capacity of storage proteins can lead to cell death characterized by iron-dependent lipid peroxidation (Dixon et al., 2012; Nikseresht et al., 2019). This form of cell death, known as ferroptosis, occurs due to iron ions catalyzing the Fenton reaction (Deng et al., 2023), generating hydroxyl or alkoxyl radicals, thereby exacerbating cellular oxidative damage (Dixon et al., 2012). Given the high dependence of the brain on iron, the deposition of iron in the brain increases with age (Belaidi and Bush, 2016). Iron deposition in the brain primarily occurs in cortical regions and internal nuclei such as the pallidum, putamen, substantia nigra, which are associated with various neurodegenerative diseases (Ward et al., 2014).

Parkinson’s disease (PD) is a common neurodegenerative disorder characterized by motor symptoms such as resting tremor, as well as non-motor symptoms including depression, anxiety, or apathy (Hayes, 2019). The main pathological features of PD include the formation of Lewy bodies in the substantia nigra pars compacta and degeneration and death of dopaminergic neurons (Shahmoradian et al., 2019). The aggregation of α-synuclein (α-syn) into Lewy bodies is currently considered a key pathological feature of PD (Wurster et al., 2022), and α-syn has been implicated in iron-mediated cell death and lipid metabolism (Li et al., 2022). Previous observational studies have found that iron deposition in the brains of PD patients primarily occurs in the glia and dopaminergic neurons of the substantia nigra, and the amount of iron deposition is closely related to the severity of the disease (Pyatigorskaya et al., 2015). Studies have shown that iron chelators can protect neurons by inhibiting iron-mediated cell death, and animal experiments have demonstrated that the use of iron chelators in α-synuclein-aggregated mouse models can rescue behavioral deficits (Zeng et al., 2021). Therefore, maintaining the balance of iron homeostasis and inhibiting iron-mediated cell death may represent a novel therapeutic approach for treating Parkinson’s disease.

The liver is an important organ for storing and metabolizing iron in the body, and hepatocytes are the second largest iron-storing cells after red blood cells (Nemeth and Ganz, 2021). A portion of the iron absorbed by the body is stored in the liver, intestines, and macrophages in the form of ferritin, while another portion is transported to the liver via the portal vein and absorbed under the mediation of transferrin (Kohgo et al., 2008). Transferrin levels are regulated by hepcidin, a peptide secreted by hepatocytes (Camaschella et al., 2020), so the liver is typically the first organ to exhibit signs of iron overload when iron overload occurs in the body (Headley et al., 2020). Liver iron concentration is a reliable indicator for assessing body iron levels (Ang et al., 2017). Aging red blood cells are engulfed by macrophages and broken down, releasing iron, with a significant portion of the released iron being stored in the spleen (Ramm and Ruddell, 2010). Iron deposition in the pancreas occurs because pancreatic β cells express more transferrin compared to other tissues (Singh et al., 2017). Previous studies have found that changes in tissue iron levels in the liver, spleen, and pancreas are consistent with changes in serum iron (Wood et al., 2015; Karakus et al., 2017; Sussman et al., 2020). In our study, we used the iron content in liver, spleen, and pancreas tissues to represent the level of iron metabolism in the body, predicting the occurrence of Parkinson’s disease at a macro level, which facilitates monitoring of the disease progression and early intervention.

Iron dysregulation in abdominal organs may contribute to the pathogenesis of Parkinson’s disease (PD) through several potential mechanisms. One such mechanism is oxidative stress. Abdominal organs, such as the liver, can generate reactive oxygen species (ROS) through the Fenton reaction. These ROS can cause oxidative damage to cellular components, including lipids, proteins, and DNA, not only within the liver but also in distant organs like the brain. Due to its high oxygen consumption and abundant lipid content, the brain is particularly susceptible to oxidative stress, which can lead to significant damage and contribute to the neurodegenerative processes observed in PD (Ward et al., 2014).Another possible mechanism is neuroinflammation. Iron overload in abdominal organs can trigger systemic inflammation, which can cross the blood–brain barrier and induce neuroinflammatory responses (Badanjak et al., 2021). Chronic neuroinflammation is implicated in the progression of PD, leading to the degeneration of dopaminergic neurons in the substantia nigra (Tansey and Goldberg, 2010). Additionally, iron dysregulation may impact mitochondrial function in both peripheral tissues and the brain, further contributing to neuronal death and the progression of PD (Sohrabi et al., 2023).

Our study has several strengths. To our knowledge, we are the first to use two-sample Mendelian randomization (MR) technique to explore the relationship between iron levels in multiple abdominal organs (liver, spleen, and pancreas) and PD. Compared to previous observational studies, MR studies are less susceptible to confounding factors and provide more stable estimates of causal effects, making MR analysis a viable alternative research method in the absence of randomized controlled trials. Additionally, the use of large-sample GWAS summary data significantly increases statistical power compared to small-sample observational studies. Furthermore, the adoption of sensitivity analysis ensures the consistency of causal estimates and the reliability of results.

Certainly, our study also has some limitations. Firstly, the data utilized in this study are derived exclusively from European populations. As such, the generalizability of these results to other ethnic groups is uncertain. Different populations may have varying genetic backgrounds, environmental exposures, and lifestyle factors that could influence both iron metabolism and Parkinson’s disease (PD) risk. Future studies should include diverse populations to confirm the applicability of our findings across different ethnicities. Secondly, the GWAS data we used were not stratified by gender and age. Iron metabolism and PD risk may differ between males and females and across different age groups. For instance, hormonal differences, such as those related to estrogen, could influence iron homeostasis and oxidative stress levels. Age-related changes in iron accumulation and brain vulnerability to oxidative damage are also important considerations. Stratified analyses could provide more nuanced insights into how these factors interact with iron metabolism and PD risk. Thirdly, despite using sensitivity analysis to test the assumptions of MR studies, it is still unable to completely eliminate horizontal pleiotropy among instrumental variables. Horizontal pleiotropy occurs when genetic variants influence the outcome through pathways other than the exposure of interest. Further methodological advancements and rigorous sensitivity analyses are necessary to enhance the validity of MR studies.

Our MR analysis has revealed the relationship between iron levels in multiple organs and the occurrence of Parkinson’s disease at a genetic level, further confirming the important role of iron homeostasis in Parkinson’s disease. The implications of our findings for clinical practice and public health are significant. Monitoring liver iron levels could become an essential aspect of PD risk assessment, particularly for individuals with genetic predispositions to iron dysregulation. Interventions aimed at regulating iron metabolism, such as iron chelation therapy or dietary modifications, might be explored as potential strategies to reduce PD risk. By integrating genetic research, clinical practice, and public health strategies, we can better prevent and manage PD, ultimately improving patient outcomes and quality of life. Further research is needed to explore the corresponding genetic mechanisms behind this relationship.

## Data availability statement

The original contributions presented in the study are included in the article, further inquiries can be directed to the corresponding author.

## Author contributions

MY: Data curation, Formal analysis, Investigation, Writing – original draft, Writing – review & editing. CT: Data curation, Formal analysis, Investigation, Writing – original draft, Writing – review & editing. FP: Data curation, Formal analysis, Investigation, Writing – review & editing. CL: Methodology, Writing – review & editing. GC: Software, Writing – review & editing. RK: Validation, Writing – review & editing. PP: Conceptualization, Funding acquisition, Supervision, Writing – review & editing.
